# Stratégie thérapeutique devant une hyperplasie thymique massive associée à un syndrome de détresse respiratoire chez un nouveau-né dans un pays en voie de développement: à propos d’un cas

**DOI:** 10.11604/pamj.2022.43.145.35801

**Published:** 2022-11-17

**Authors:** Fabrice Stéphane Arroye Betou, Souleymane Diatta, Papa Amath Diagne, Moussa Seck Diop, Kondo Bignandi, Gabriel Ciss, Assane Ndiaye

**Affiliations:** 1 Université de Cheikh Anta Diop, Faculté de Médecine, de Pharmacie et d’Odontologie, Centre Hospitalier Universitaire de Fann, Service de Chirurgie Thoracique et Cardiovasculaire, Dakar, Sénégal; 2 UFR des Sciences de la Santé, Ziguinchor, Sénégal.

**Keywords:** Hyperplasie thymique, chirurgie, détresse respiratoire, cas clinique, Thymic hyperplasia, surgery, respiratory distress, case report

## Abstract

L´hyperplasie thymique est une masse médiastinale antérieure dont le tableau clinique est varié, qui pose chez le nourrisson de réels problèmes diagnostiques et pour laquelle la stratégie thérapeutique ne fait l´objet d´aucun consensus. Nous rapportons le cas d´un nourrisson d’un mois, suivi pour un syndrome de détresse respiratoire. La tomodensitométrie thoracique a révélé une masse médiastinale antérieure, qui a été retirée par sternotomie médiane. L´examen anatomopathologique était en faveur d´une hyperplasie thymique vraie et l´évolution clinique était satisfaisante. Au vu de ce résultat encourageant, et contrairement à ce que proposent certains auteurs, il serait plus opportun d´opter d´emblée pour une stratégie thérapeutique agressive, lors de la prise en charge d´une hyperplasie thymique symptomatique. Ceci se justifie d´autant plus dans un contexte socio-économique caractérisé par un accès difficile aux soins et des mesures de suivi limitées par les moyens des patients.

## Abstract (en)

Thymic hyperplasia is an anterior mediastinal mass with a variable clinical presentation. It causes differential diagnostic problems in the pediatric age group and there is no consensus on the therapeutic approach. We here report the case of a 1-month-old infant treated for respiratory distress syndrome. Chest CT scan revealed anterior mediastinal mass, which was excised through median sternotomy. Anatomopathological examination showed thymic hyperplasia. Clinical outcome was satisfactory. This encouraging result suggests that, contrary to what some authors propose, it would be more appropriate to opt for an aggressive therapeutic strategy when managing symptomatic thymic hyperplasia. This is even more justified in a socio-economic context characterised by difficult access to care and follow-up measures limited by patients’ means.

## Introduction

Les hyperplasies thymiques vraies sont des pathologies pseudo-tumorales bénignes de la glande thymique. Elles sont rares et constituent 1% de l´ensemble des lésions du thymus [1]. Généralement asymptomatiques, elles ne présentent que très peu de complications, et peuvent être traitées médicalement lorsqu´un diagnostic de certitude est posé. Cependant, comme cela a été décrit par Linegar *et al*., elles peuvent être diagnostiquées chez un patient présentant des symptômes résultant d´une compression des voies aériennes respiratoires et du parenchyme pulmonaire [2]. Devant les difficultés diagnostiques face aux autres tumeurs du médiastin antérieur, il existe une controverse au sujet de l´attitude thérapeutique la plus idoine, notamment chez le nouveau-né. Nous vous rapportons le cas d´une hyperplasie thymique du nouveau-né traitée dans notre service.

## Patient et observation

**Information relative au patient:** un nouveau-né d´un mois nous avait été adressé du service de pédiatrie d´un centre hospitalier en zone rurale pour la prise en charge d´une masse médiastinale antérieure. Il était issu d´un accouchement à terme et par voie basse, eutocique, avec un score d´APGAR supérieur à 7, au terme d´une grossesse bien suivie. Son poids à la naissance était de 3800 grammes, et il était sous allaitement maternel exclusif.

**Figure 1:**
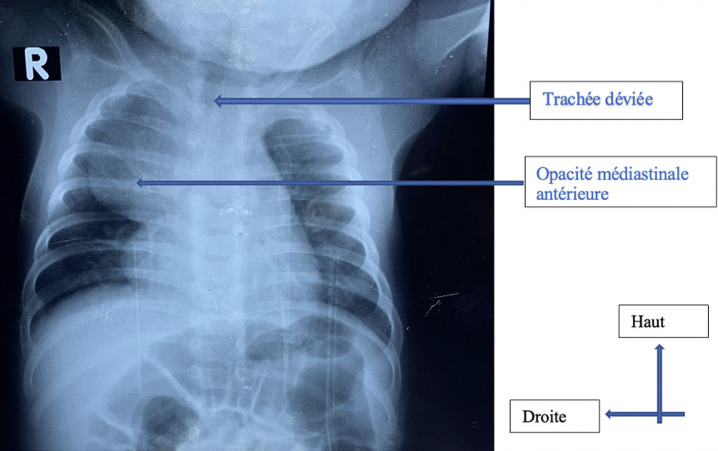
radiographie du thorax

**Figure 2:**
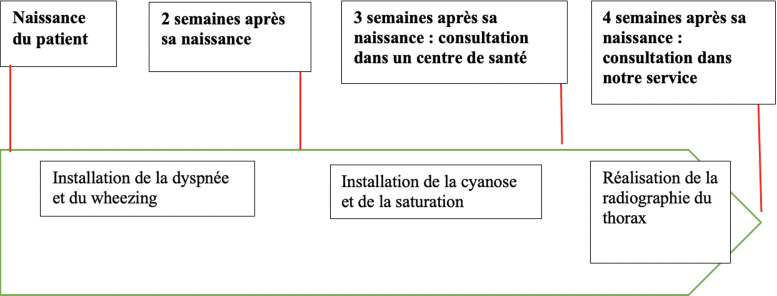
frise chronologique de l´histoire de la maladie

**Résultats cliniques:** il présentait une symptomatologie faite de dyspnée intermittente inspiratoire, sans horaire, d´installation progressive, et se majorant avec le temps, associée à un wheezing, un tirage sus-sternal, une cyanose, avec une saturation à l´air ambiant conservée. Cette symptomatologie évoluait depuis 2 semaines après sa naissance. Devant ce tableau, une radiographie du thorax avait été réalisée. Elle avait permis de mettre en évidence une opacité médiastinale antérosupérieure, associée à une légère déviation trachéale à droite (Figure 1). Le patient nous avait par la suite été adressé. Une frise chronologique sur la Figure 2 illustre les différents temps de l´histoire de la maladie du patient. Cliniquement, à son admission, il présentait en plus de son syndrome de détresse respiratoire, une saturation pulsée à 93% à l´air ambiant.

**Figure 3:**
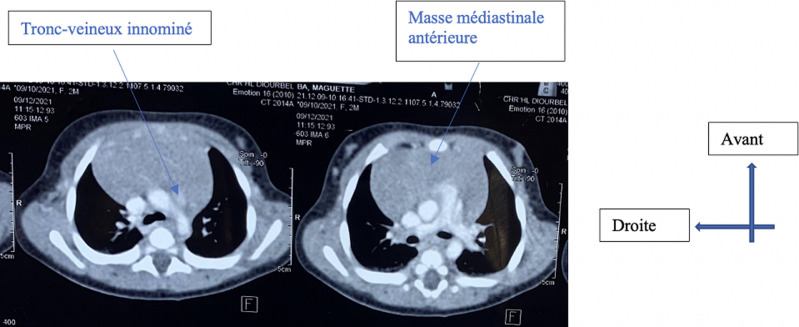
tomodensitométrie thoracique: coupes transversales

**Figure 4:**
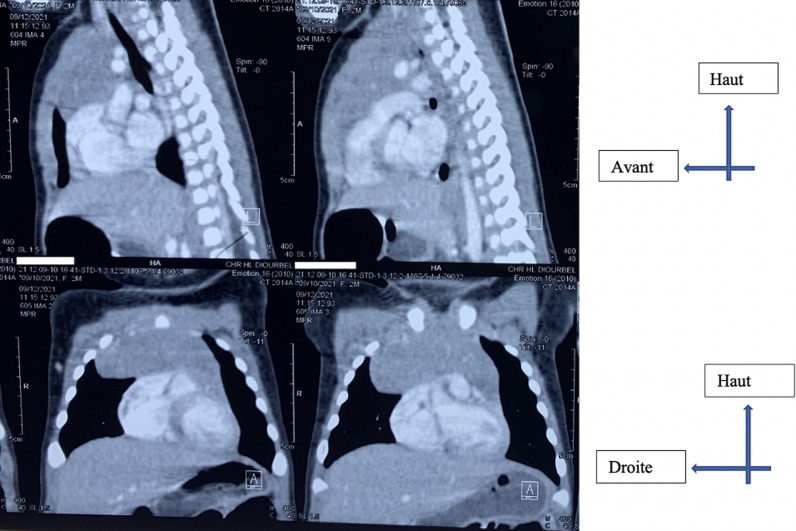
tomodensitométrie thoracique: coupes sagittales et coronales

**Démarche diagnostique:** une tomodensitométrie thoracique avait par la suite été réalisée, ayant permis de mettre en évidence une masse médiastinale antérieure, de densité tissulaire, développée aux dépens du thymus, avec comme mensurations 56 mm x 52 mm de grand axe. Elle exerçait un effet de masse sur le parenchyme pulmonaire gauche et droit (Figure 3 et Figure 4).

Afin d´exclure parmi les diagnostics différentiels une tumeur germinale non séminomateuse, les dosages de l´hormone gonadotrophine chorionique humaine, ainsi que l´alpha-fœto-protéine ont été effectués, et sont tous revenus normaux. Le dosage des lactate deshydrogénase (LDH) avait également été effectué et était également revenu normal. Celui des anticorps anti-récepteurs de l´acétylcholine n´avait par contre pas été réalisé du fait des difficultés financières de la famille du patient. Sur la base de ces éléments cliniques et paracliniques, le diagnostic d´une tumeur épithéliale thymique a été évoqué. Nous ne pouvions formellement exclure d´autres diagnostics différentiels tels qu´un lymphome, ou un séminome qu´après une confirmation anatomopathologique de cette tumeur, à la suite de l´exploration chirurgicale de cette masse, qui nous a semblé du reste extirpable au vu des images tomodensitométriques. Les différentes investigations réalisées pour exclure les diagnostics différentiels sont résumées dans le Tableau 1.

**Tableau 1:** diagnostics différentiels de l´hyperplasie thymique

Diagnostics différentiels	Éléments recherchés pour l’exclure
Tératome mature ou immature	**Tomodensitométrie:** pas de densité hétérogène, absence de remaniement adipeux, kystique et ou hémorragique.
Tumeurs germinales non séminomateuses	**Biologie:** BHCG et alpha fœto-protéines négatives
Séminome	
Lymphome non hodgkinien ou maladie de Hodgkin	**Clinique:** pas d’apparition rapide de signe B, pas d’adénopathie périphérique. **Biologie:** pas d´élévation de taux sériques de lactates déshydrogénase.
Carcinome thymique	Exploration chirurgicale pour confirmation anapath
Hyperplasie thymique	Exploration chirurgicale pour confirmation anapath
Thymome	Exploration chirurgicale pour confirmation anapath

BHCG human chorionic gonadotrophin de type béta

**Figure 5:**
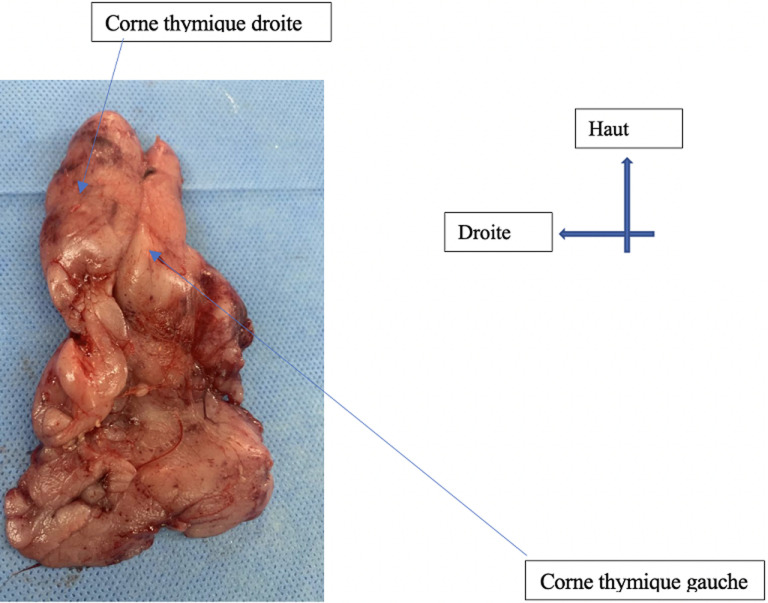
pièce opératoire de thymectomie radicale

**Figure 6:**
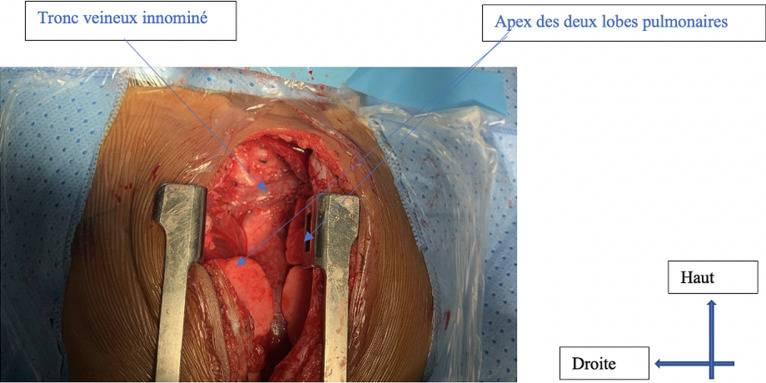
loge thymique évidée après exérèse du thymus

**Intervention thérapeutique:** après prélèvement du bilan biologique pré-opératoire, l´indication opératoire d´une thymectomie radicale par sternotomie médiane a été posée. L´exploration per-opératoire avait révélé une masse thymique souple, non hémorragique, et homogène, d´environ 50 grammes. Elle mesurait 9 x 5 x 3 cm, et adhérait au plan profond. Elle était en rapport avec le péricarde, la plèvre médiastinale droite, et gauche, la veine cave supérieure, et le tronc veineux brachio-céphalique droit qu´elle engainait sur sa face antérieure, avec un plan de clivage (Figure 5). Il avait par conséquent été réalisé comme geste une exérèse du thymus ainsi qu´une portion des franges péricardiques et de la graisse thymique. Les nerfs phréniques droit et gauche avaient été vus et respectés, et la loge thymique totalement évidée (Figure 6). L´ouverture accidentelle des deux plèvres droite et gauche était survenue comme incident per-opératoire. Elle avait motivé la fermeture de la cavité thoracique sous trois drains thoraciques, dont un rétro-sternal et deux pleuraux.

**Suivi et résultats:** les suites opératoires ont été simples, et le séjour en hospitalisation a été de 8 jours. Il a été observé une nette amélioration de l´état clinique du malade avec une régression complète de la détresse respiratoire. Les radiographies de contrôle à M3 et M6 post-opératoire sont toutes revenues normales. Les résultats de l´examen anatomopathologique sont revenus en faveur d´une hyperplasie thymique. Un suivi annuel lui a été prescrit.

**Consentement éclairé:** les parents du patient considérés comme ses tuteurs légaux, ont donné leur consentement éclairé.

## Discussion

Les hyperplasies thymiques constituent une ensemble de pathologies rares, à partir desquelles, sur la base des critères histologiques et macroscopiques, notamment la présence et la proportion relative du contingent des cellules folliculaires lymphoïdes avec des centres germinatifs, la taille et la masse de la glande, on distingue 2 types: celui dit vrai, et celui lympho-folliculaire. L´hyperplasie vraie est caractérisée par une augmentation de la taille et du poids du thymus, qui conserve une morphologie normale ou presque normale pour le patient, et n´est pas accompagnée de follicules lymphoïdes avec des centres germinaux activés. Parmi les hyperplasies vraies, ont été décrites les hyperplasies thymiques massives idiopathiques, et les hyperplasies thymiques rebond. Celles massives idiopathiques se définissent comme étant des hypertrophies du cortex et de la médullaire de la glande thymique, sans anomalie histologique, associées à une augmentation de la masse du thymus, supérieure à l´ombre cardiaque sur le télécoeur radiologique de face, et/ou par un poids du thymus supérieur à 2% de la masse corporelle [3].

La présentation clinique des hyperplasies thymiques chez les enfants est variable. Elles peuvent être asymptomatiques (38%) ou présenter des signes de détresse respiratoire (29%), des infections pulmonaires (35%), une toux ou une dysphagie [4]. Ces signes de détresse respiratoire s´expliquent généralement par une compression trachéale exercée par la masse. Devant une masse médiastinale antérieure développée aux dépens du thymus chez un nourrisson, établir un diagnostic différentiel d´hyperplasie thymique est difficile, car un thymome, voir un carcinome thymique peuvent être évoqués. Elle peut éventuellement être envisagée après un stress, des brûlures, l´administration d´une chimiothérapie, d´une radiothérapie, d´un traitement antihormonal, ou d´un traitement aux stéroïdes. Ceci fait intervenir le concept de rebond thymique, autre sous-type d´hyperplasie thymique vraie, cité plus haut. L´importance de la reconnaissance de la nature du rebond thymique réside dans le fait qu´une fois son diagnostic établi, aucun traitement spécifique n´est nécessaire. Ceci contraste avec l´hyperplasie thymique massive vraie, où il existe une controverse en ce qui concerne une prise en charge éventuellement chirurgicale.

Bien que le diagnostic positif d´une hyperplasie thymique repose essentiellement sur l´examen anatomopathologique, elle présente des caractéristiques tomodensitométriques spécifiques tels que décrites par Priola *et al*. [5], à savoir une lésion médiastinale antérieure respectant la forme bipyramidale triangulaire, symétrique du thymus normal, avec une densité homogène et une densité mixte, graisseuse de faible atténuation. L´imagerie par résonnance magnétique, utilisant des séquences en contraste de phase, pourrait également permettre une identification de cette infiltration graisseuse microscopique typique des hyperplasies, qui n´est pas observée en cas de lésion tumorale [6]. En ce qui concerne le cas de nôtre étude, nous relevons que les images tomodensitométriques du patient ne présentaient pas l´aspect typique décrit dans la littérature de lésion thymique hyperplasique, mais plutôt un aspect asymétrique, de forme irrégulière. Ce cas nous permet ainsi de mettre en exergue les difficultés diagnostiques auxquelles cette pathologie nous confronte. Comme en ce qui concerne le diagnostic des tumeurs épithéliales thymiques, la tomographie par émission de positons au 18-fluoro-désoxyglucose n´a que très peu d´intérêt dans la prise en charge diagnostique des hyperplasies thymiques. En effet, même si leur métabolisme est généralement inférieur à celui des tumeurs de grade plus élevés tels que les thymome B3 et les carcinomes thymiques, les valeurs prédictives positives et négatives et le seuil optimal des valeurs d´absorption sont variables d´une étude à l´autre. En outre des hyperplasies thymiques peuvent être associées à des niveaux d´hyper métabolisme élevés [7].

Une cytoponction à l´aiguille ou une biopsie scanno-guidée peuvent être réalisées, comme l´ont rapporté Riazmontazer *et al*. [8]. En cas de doute, une biopsie pré thérapeutique par voie percutanée, chirurgicale par médiastinotomie ou mini-thoracotomie pourraient être envisagées. En revanche, elle n´est pas nécessaire si le diagnostic de tumeur épithéliale thymique est fortement suspecté et si l´exérèse chirurgicale est réalisable d´emblée, ce qui était le cas chez nôtre patient. Certains auteurs tels que Mlika *et al*. [9] préconisent une corticothérapie première à base de prednisone par voie orale à la dose de 60 mg/mg/m^2^ pendant 7 à 10 jours chez les enfants. Cette attitude est motivée par la prévention des effets néfastes de la thymectomie sur cette tranche d´âge. Partant de ce postulat, Kent *et al*. [10] ont suggéré que les thymus normaux sont peut-être trop souvent retirés lorsqu´un patient ne présente aucun autre problème qu´une glande qui est ressentie comme légèrement agrandie. Selon Kaira *et al*. [11], le diamètre d´une lésion supérieur à 30 mm, constitue le seuil au-delà duquel la possibilité d´une tumeur thymique évolutive est suffisamment élevée pour justifier une prise en charge diagnostique. Ceci a amené Girard *et al*. à préconiser une abstention thérapeutique devant une hyperplasie thymique avec un diamètre inférieur à 30 mm [12]. Encore qu´il faudrait effectivement avoir établi le diagnostic de certitude de l´hyperplasie thymique.

Le diamètre de la lésion thymique chez nôtre patient ayant été mesuré à plus de 5 cm, une indication chirurgicale pouvait effectivement se discuter. Cette attitude thérapeutique est en outre appuyée par Diarra *et al*., qui avaient recommandé une thymectomie d´emblée, dans des contextes de pratique marquée par un suivi médical irrégulier et incertain [3]. Tous ces éléments sus-évoqués doivent être pris en compte avant de recommander une intervention chirurgicale à une personne ayant un thymus “élargi”. Malheureusement, personne n´a jamais réalisé d´étude définitive établissant ce qui constitue une taille thymique “normale” à différents âges, ceci constituant une des limites objectives de notre travail. Le chirurgien thoracique est donc généralement laissé à son jugement et à son expérience pour décider si le thymus d´un patient référé est normal ou hypertrophié.

## Conclusion

Ce cas, pour lequel l´option thérapeutique chirurgicale a été de rigueur, nous permet de faire ressortir les difficultés liées à la prise en charge de l´hyperplasie thymique et nous met en face d´un vrai dilemme. Ceci pourrait nous permettre de revoir et discuter d´une codification thérapeutique bien clarifiée de ce groupe de pathologies, quel que soit le contexte sous lequel nous nous trouvons.
